# Instruments for assessing foot self-care of people with diabetes: a scoping review

**DOI:** 10.1590/0034-7167-2022-0555

**Published:** 2023-08-07

**Authors:** Amelina de Brito Belchior, Florência Gamileira Nascimento, Mariane Carlos de Sousa, Ana Beatriz Mesquita da Silveira, Sherida Karanini Paz de Oliveira

**Affiliations:** I Universidade Estadual do Ceará. Fortaleza, Ceará, Brazil; II Universidade de Fortaleza. Fortaleza, Ceará, Brazil

**Keywords:** Diabetic Foot, Self Care, Questionnaire, Diabetes Complications, Nursing, Pie Diabético, Autocuidado, Cuestionario, Complicaciones de la Diabetes, Enfermaría, Pé Diabético, Autocuidado, Questionário, Complicações do Diabetes, Enfermagem

## Abstract

**Objectives:**

to map, in the world literature, instruments for assessing foot self-care of people with diabetes.

**Methods:**

a scoping review in Scopus by Elsevier, MEDLINE via PubMed, LILACS, SciELO databases and gray literature, using the controlled words diabetic foot, self care, questionnaire. Search was carried out in February and March 2021, according to JBI recommendations and PRISMA-ScR extension.

**Results:**

fifteen studies made up the review, 14 articles and one thesis, published between 2000 and 2020. 16 instruments were identified: five with an emphasis on general self-care and 11 on foot self-care. Inspection, hygiene, washing and drying between the toes, lotion application and use of proper shoes and socks were the main self-care measures presented.

**Final Considerations:**

foot self-care is assessed by knowledge, social support and frequency with which measures are being put into practice, encouraging professional practice.

## RESUMO

**Objetivos:**

mapear, na literatura mundial, instrumentos de avaliação do autocuidado com os pés de pessoas com diabetes.

**Métodos:**

revisão de escopo nas bases de dados Scopus da Elsevier, MEDLINE via PubMed, LILACS, SciELO e literatura cinzenta, utilizando os vocábulos controlados *diabetic foot, self care, questionnaire* . Busca foi realizada em fevereiro e março de 2021, conforme as recomendações do JBI e extensão do PRISMA-ScR.

**Resultados:**

15 estudos compuseram a revisão, sendo 14 artigos e uma tese, publicados entre 2000 e 2020. Foram identificados 16 instrumentos: cinco com ênfase no autocuidado geral e 11 no autocuidado com os pés. Inspeção, higiene, lavagem e secagem entre os dedos, aplicação de loção e uso de calçados e meias propriados foram as principais medidas de autocuidado apresentadas.

**Considerações Finais:**

o autocuidado dos pés é avaliado pelo conhecimento, apoio social e frequência com que as medidas estão sendo colocadas em prática, fomentando a prática profissional.

## RESUMEN

**Objetivos:**

mapear, en la literatura mundial, instrumentos para evaluar el autocuidado con los pies de personas con diabetes.

**Métodos:**

revisión de alcance en las bases de datos Scopus de Elsevier, MEDLINE vía PubMed, LILACS, SciELO y literatura gris, utilizando las palabras controladas *diabetic foot, self care, questionnaire* . La búsqueda se realizó en febrero y marzo de 2021, según recomendaciones del JBI y extensión de PRISMA-ScR.

**Resultados:**

15 estudios integraron la revisión, 14 artículos y una tesis, publicados entre 2000 y 2020. Se identificaron 16 instrumentos: cinco con énfasis en el autocuidado general y 11 en el autocuidado con los pies. La inspección, higiene, lavado y secado entre los dedos, aplicación de loción y uso de zapatos y calcetines adecuados fueron las principales medidas de autocuidado presentadas.

**Consideraciones Finales:**

el autocuidado de los pies se evalúa por el conocimiento, el apoyo social y la frecuencia con la que se están poniendo en práctica las medidas, incentivando la práctica profesional.

## INTRODUCTION

Diabetic foot ulcer (DFU) is the most frequent chronic complication in people with Diabetes Mellitus (DM). Of multifactorial etiology, it is a necro suppurative process and/or destruction of soft tissues, associated with diabetic neuropathy and peripheral arterial disease (PAD) of the lower extremities^( 1 )^. Its incidence varies between 2-4%, with a prevalence of 4-10%^( 2 )^.

According to the International Diabetes Federation (IDF), estimates show that every 20 seconds a lower extremity is amputated due to diabetes complications. This fact can be proven by the estimate that 25% of people with diabetes will develop at least one DFU throughout their lives^( 3 )^. In Brazil, the increasing number of lower extremity amputations due to diabetes complications, performed by the Unified Health System (SUS – *Sistema Único de Saúde* ), until September 2021, showed an increase of 4.18% compared to the previous year^( 4 )^.

DFU is the most common cause of amputations of toes and lower extremities, and is accentuated in the presence of obesity, immunological deficiency and PAD^( 5 )^. This fact has repercussions on a person’s personal life and self-perception, affecting their self-esteem and self-image, triggering feelings such as fear, shame, frustration and impotence in the face of the limitation of their role in the family and social context. This makes a person more prone to depression, whose factors are related to the presence and fear of complications^( 6 , 7 , 8 )^.

Thus, health education actions associated with a multifactorial clinical approach, respecting each person’s characteristics, are resources capable of encouraging self-care and identifying risk factors, with a view to preventing complications such as DFU^( 9 )^. Self-care, daily foot self-examination and clinical foot examination are low-cost, simple and effective primary preventive measures that provide early detection and timely treatment of changes^( 9 , 10 )^.

A recent integrative review showed that non-compliance with self-care for the feet of people with diabetes is related to a lack of knowledge about this activity and the inability of some nursing professionals to carry out care guidelines that promote adherence by this public^( 11 )^.

Therefore, it is essential to monitor self-care actions through using validated assessment instruments to obtain reliable and useful data. Moreover, it enables the assessment of a person’s responses to treatment, identifying problems and needs and directing care plan, decision-making and clinical management^( 12 )^.

## OBJECTIVES

To map, in the world literature, instruments for assessing foot self-care of people with diabetes that are validated and available in the literature.

## METHODS

### Ethical aspects

To carry out this study, all ethical precepts were respected. All authors of the analyzed articles were properly referenced, in accordance with Copyright Law 9.610 of February 19, 1998^( 13 )^. The research data and information were presented in a reliable manner.

### Theoretical-methodological framework

#### Study design

This is a scoping review, conducted in five stages: research question identification; relevant study identification; study selection; data categorization and collection; and synthesis and mapping of results^( 14 )^. This type of literature review is aimed at mapping the main concepts and limitations of a given area of research as well as evidence for professional practice, guided by the JBI Institute Reviewer’s Manual assumptions^( 14 )^.

#### Step 1: Guiding question identification

For a better presentation, the PRISMA Extension for Scoping Reviews (PRISMA-ScR): Checklist and Explanation recommendations were used^( 15 )^.

The PCC strategy (Population, Concept and Context)^( 16 )^ was used to formulate the guiding question: which validated instruments for assessing foot self-care of people with diabetes are available in the literature?

#### Step 2: Study setting

The studies were identified through an electronic search in primary and secondary sources and in gray literature, published in any language, with no time restriction, which included validated foot self-care assessment instruments or which in some dimension demonstrated such care. Manuals, instructions and studies that did not contemplate the guiding question or did not present a reference to the instrument were excluded.

#### Step 3: Data collection and organization

The search took place from February to March 2021 in the following databases, repositories and directories: Scopus by Elsevier; MEDLINE (National Library of Medicine) via PubMed; LILACS (Latin American and Caribbean Literature in Health Sciences); SciELO (Scientific Electronic Library Online); CAPES Catalog of Theses and Dissertations; ProQuest Dissertations and Theses (PQDT); Brazilian Digital Library of Theses and Dissertations; and Google Scholar.

For the search strategy, controlled Medical Subject Heading (MeSH) and Health Sciences Descriptors (DeCS) descriptors were used, in addition to keywords, to expand the material available in the literature. Furthermore, Boolean operators OR and AND were used, in addition to opting for similar terms present in MeSH and DeCS. It is worth mentioning that the search strategies were combined according to each database, considering the descriptors “diabetic foot”, “self care”, “questionnaire” ( Chart 1 ).

**Chart 1:** Search strategies according to each database, Fortaleza, Ceará, Brazil, 2022

Database	Strategy	Total studies found
MEDLINE	(diabetic foot) OR (foot ulcer diabetic) AND (self care) OR (Selfcare) OR (self-care) AND (Questionnaire) OR (Surveys)	442
LILACS		50
SciELO	( *pé diabético* ) AND ( *autocuidado* ) AND ( *questionários* ) OR ( *instrumentos* )	24
Scopus	“diabetic foot” AND “self care” AND “questionnaire”	333
PubMed	(((“Diabetic foot” OR “Diabetic Feet” OR “Foot Ulcer, Diabetic”)) AND ((“Self care” OR “Self-Care” OR “SelfCare”))) AND ((“Surveys and Questionnaires” OR “Questionnaires and Surveys” OR “Survey Methods” OR “Survey Method” OR “Surveys” OR “Questionnaire Design” OR “Questionnaire Designs” OR “Questionnaires” OR “Questionnaire”))	104
Google Scholar		92
CAPES catalog	(diabetic food) OR (foot ulcer diabetic) AND (self care) OR (Selfcare) OR ( self-care) AND (Questionnaire) OR (Surveys)	47
BBDTD	“( *Todos os campos*: ( *Pé Diabético* ) OR ( *Úlcera Diabética do* Pé *)* OR ( *neuropatia diabética* ) *E Todos os campos*: ( *Autocuidado* ) OR ( *Autoajuda* ) *E Todos os campos*: ( *Inquéritos e Questionários* ) OR ( *Questionário* ) OR ( *Questionários* ) OR ( *Inquéritos* ))”	11
ProQuest	(diabetic foot) AND (self care) AND (Questionnaire) AND ti(diabetic food AND self care AND questionnaire	15
TOTA L		1,118

#### Step 4: Data analysis

Two examiners independently participated in study eligibility, using the software Endnote web ( https://www.myendnoteweb.com/EndNoteWeb.html ) and Excel spreadsheets for managing the studies. Initially, the examiners performed a screening based on reading the titles and abstracts. Afterwards, in a consensus meeting, article selection was confirmed, justifying the exclusion according to the established criteria. At this stage, studies were read in full, and then references were analyzed for inclusion of new studies. To identify gray literature, we chose to search specific databases for theses and dissertations.

Data extraction and management were carried out through mapping, containing characterization information, such as authorship, instrument name, objective, country of origin, methodological aspects; and instrument characterization: type, objectives, domains, dimensions, items, form of assessment and psychometric data. At the end, a critical summary was elaborated, synthesizing all this information.

## RESULTS

**Figure 1:**
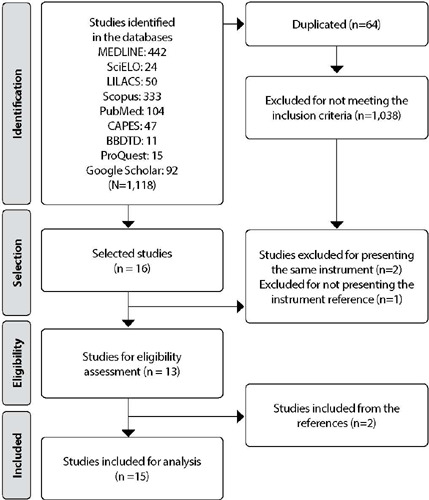
PRISMA flowchart for selecting scoping review studies, Fortaleza, Ceará, Brazil, 2022

A total of 1,118 studies were found, 1,045 of which were in the databases and 73 in gray literature for the selection analysis process. After removing the duplicates (64), 1,041 were excluded after reading the title and abstract, because they did not mention which instrument was used in the study or because it was an instrument already selected. Soon after, 16 studies were screened to be read in full. At the end of this analysis, 13 eligible studies were shown and included in the sample. After checking the references of these studies, two more studies were identified, totaling 15 ( Figure 1 ).

Of the 15 selected studies^( 17 , 18 , 19 , 20 , 21 , 22 , 23 , 24 , 25 , 26 , 27 , 28 , 29 , 30 , 31 )^, 14 were articles^( 17 , 18 , 19 , 20 , 21 , 23 , 24 , 25 , 26 , 27 , 28 , 29 , 30 , 31 )^ and one was a thesis^( 22 )^, published between 2000 and 2020. The countries of origin were well distributed across three continents, with three in Asia^( 21 , 24 , 31 )^, seven in Europe^( 17 , 18 , 23 , 25 , 28 , 29 , 30 )^ and five in the Americas^( 19 , 20 , 22 , 26 , 27 )^, with the majority in Germany^( 18 , 25 )^ and Brazil^( 19 , 22 )^ (two studies each).

Six studies were identified, which presented original instruments^( 21 , 22 , 24 , 26 , 28 , 30 )^. Five are cross-cultural adaptations to other languages^( 18 , 19 , 23 , 27 , 29 )^, two are cross-sectional studies^( 20 , 23 )^, one is a quasi-experimental study^( 31 )^, and one is a review of reliability, validity and data^( 17 )^.

The Summary of Diabetes Self-Care Activities (SDSCA) instrument was found in three studies in English (original)^( 17 )^, German^( 18 )^ and Brazilian Portuguese^( 19 )^; The diabetic foot self-care questionnaire of the University of Malaga (DFSQ-UMA), in Spanish (original)^( 28 )^ and French^( 29 )^; and the Foot Care Confidence Scale (FCCS), in English (original)^( 26 )^ and Mexican Spanish^( 27 )^. Thus, regarding the instruments, five general self-care instruments were found^( 17 , 18 , 19 , 20 , 21 )^ and 11 specific self-care instruments for the feet^( 22 , 23 , 24 , 25 , 26 , 27 , 28 , 29 , 30 , 31 )^ ( Chart 2 ).

**Chart 2:** Characterization of foot self-care scoping review studies, Fortaleza, Ceará, Brazil, 2022

Title	Journal/year	Country of origin	Study design	Instrument
**General self-care instruments, including foot care**				
The summary of diabetes self-care activities measure: results from 7 studies and a revised scale^( 17 )^	Diabetes Care/2000	United Kingdom	Review of reliability, validity and data	Summary of Diabetes Self-Care Activities (SDSCA)
Assessing self-management in patients with diabetes mellitus type 2 in Germany: validation of a German version of the Summary of Diabetes Self-Care Activities measure (SDSCA-G)^( 18 )^	Health and Quality of Life Outcomes/2014	Germany	Instrument translation methodology	Version of the Summary of Diabetes Self-Care Activities measure (SDSCA-G)
*Questionário de Atividades de Autocuidado com o Diabetes: tradução, adaptação e avaliação das propriedades psicométricas* ^( 19 )^	*Arquivos Brasileiros de Endocrinologia & Metabologia/2010*	Brazil	Instrument translation methodology	Questionário de Atividades de Autocuidado com o Diabetes (QAD)
The capabilities and activities of self-care in patients with diabetic foot^( 20 )^	*Revista Enfermería Herediana* /2014	Peru	Cross-sectional	Questionnaire on skills and self-care activities of patient with diabetic foot
Development andpsychometric properties ofa new social support scale for self-care in middle-aged patients with type II diabetes (S4-MAD)^( 21 )^	BMC PublicHealth/2012	Iran	Instrument preparation methodology	The social-supportscale for self-care in middle-aged patientswith type 2 diabetes (S4-MAD)
**Foot-specific self-care instruments**				
*Adesão ao autocuidadoc om os pés em diabéticos: desenvolvimento de um instrumento embasado nat eoria da resposta ao item* (TRI)^( 22 )^	*Plataf ormaSucupira* /2014	Brazil	Instrument preparation methodology	*Questionário de Atividades de Autocuidado com osPés para Diabéticos* (QPED)
*Cuestionario sobre el comportamiento planeado en la diabetes - cuidado conlos pies: validación* ^( 23 )^	Online Brazilian Journal of Nursing/2015	Portugal	Instrument translation methodology	*Questionário do Comportamento Planeado na Diabetes – Cuidado Com os Pés* (QCP-CP)
Development andv alidation of a diabetesf oot self-care behavior scale^( 24 )^	The Journal ofNursing Research/2013	China	Instrument preparation methodology	Diabetes foot self-care behavior scale(DFSBS)
Diabetes foot self-care practices in the German population^( 25 )^	Journal ofClinical Nursing/2008	Germany	Cross-sectional	Frankfurter Catalogue of Foot Self-Care - Prevention of the Diabetic Foot Syndrome (FCFSP)
Developing and testing of the Foot Care Confidence Scale^( 26 )^	Journal of Nursing Measurement/2002	USA	Instrument preparation methodology	Foot Care Confidence Scale(FCCS)
Validity of the Mexican version of the combined Foot Care Confidence/Foot-Care Behavior scale for diabetes^( 27 )^	*Revista Panamericana de Salud Pública* /2015	Mexico	Instrument translation methodology	Foot Care Confidence Scale/Foot-Care Behavior (FCCS-FCB)
Development, validationand psychometric analysis of the diabetic foot self- care questionnaire of the University of Malaga,Spain (DFSQ-UMA)^( 28 )^	Journal of Tissue Viability/2015	Spain	Instrument preparation methodology	The diabetic foot self-care questionnaire of the University of Malaga (DFSQ- UMA)
Cross-cultural Adaptation and Validation of the French Version of the Diabetic Foot Self-care Questionnaire of theUniversity of Malaga^( 29 )^	Journal of the American Podiatric Medical Association/2019	France	Instrument translation methodology	The diabetic foot self-care questionnaire of the University of Malaga, French (DFSQ-UMA Fr)
Quality of Foot Care Among Patients With Diabetes: A Study Using a Polish Version of the Diabetes Foot Disease and Foot Care Questionnaire^( 30 )^	The Journal of Foot & Ankle Surgery/2020	Poland	Instrument preparation methodology	Diabetes Foot Disease and Foot Care (DFDFC-Q)
The effect of a foot care camp on diabetic foot care knowledge and the behaviours of individualswith diabetes mellitus^( 31 )^	Journal of Research in Nursing/2018	Indonesia	Quasi-experimental	Modified DiabeticF oot Care Knowledge (MDFK) Modified DiabeticF oot Care Behaviors(MDFCB)

The articles were grouped into two categories: 1 - Instruments that assess general DM self-care with items that assess foot self-care (5)^( 17 , 18 , 19 , 20 , 21 )^; 2 - Instruments that assess diabetic foot self-care (11)^( 22 , 23 , 24 , 25 , 26 , 27 , 28 , 29 , 30 , 31 )^.

The instruments that assess DM self-care in general and that present foot self-care items are: SDSCA^( 17 )^, Questionnaire on skills and self-care activities of patient with diabetic foot^( 20 )^ and The social-support scale for self-care in middle-aged patients with type 2 diabetes (S4-MAD)^( 21 )^. These instruments have the following dimensions in common: general diet; specific diet; exercises; blood glucose test; foot care; cigarette use; medications. In addition to these, the medical care dimension was adopted in the Questionnaire on skills and self-care activities of patients with diabetic foot^( 20 )^.

Regarding the foot care subscale, the instruments addressed common issues related to inspection, hygiene and use of appropriate footwear. Additionally, the S4-MAD contemplates daily self-care^( 20 )^, and the SDSCA, drying between the toes after washing the feet^( 17 )^. This represents a weakness of these instruments, since foot self-care is limited and assessed in an elementary way.

As for the specific foot self-care assessment instruments, there is a similarity in the approach of its dimensions through items. It is noteworthy that most instruments address foot inspection, washing, drying, finger inspection, lotion application, and shoe and sock use.

Several constructs related to diabetic feet were identified, such as preventive and risk behaviors as well as self-efficacy (Foot Care Confidence Scale/Foot-Care Behavior (FCCS-FCB))^( 27 )^; patient self-control regarding assessed dimensions (Frankfurter Catalog of Foot Self-Care – Prevention of the Diabetic Foot Syndrome (FCFSP))^( 25 )^; social support for self-care in middle-aged patients with type II diabetes (S4-MAD)^( 21 )^. Furthermore, one study presented two instruments: one assesses foot self-care knowledge before interventions (Modified Diabetic Foot Care Knowledge (MDFCK)), and the other assesses foot self-care behavior after interventions (Modified Diabetic Foot Care Behavior ( MDFCB))^( 31 )^.

There were eight questionnaires and five scales. It was found that cross-cultural adaptation studies did not perform the Content Validity Index (CVI), as it is an instrument that has already been validated. The CVI was explicitly present in only three studies, one of which used the Kappa coefficient, also presented by two other studies. For the correlation between items, Pearson’s r (N=4), Spearman’s r (N=2) and the Chi-Square tests (N=1) were used.

As for reliability, for internal consistency analysis, we used Cronbach’s alpha (N=11) and the Kuder-Richardson test (N=1). It was evident that three instruments had moderate reliability, an average of 0.651, namely DDFFC-Q, QCP-CP and the SDSCA German version, while the rest had excellent reliability rates (average = 0.842).

The only instrument that did not present the results of the tests performed was the Questionnaire on skills and self-care activities of patient with diabetic foot, which mentioned the performance of statistical tests, but omitted their results, representing a study limitation and instrument.

**Chart 3:** Characterization of instruments found in the scoping review, Fortaleza, Ceará, Brazil, 2022

Instrument name	Dimensions and items	Answer	Language	Psychometric measures
Summary of DiabetesSelf-Care Activities (SDSCA)^( 17 )^ Version of the Summary of Diabetes Self-Care Activities measure (SDSCA-G)^( 18 )^	- General diet - Specific diet - Exercises - Blood glucose test - Foot care - Cigarette use - Medications 11 items	Likert	English	Correlation: r =0.2 - 0.76
			Version German from SDSCA	Cronbach’s alpha = 0.607 Correlation: Rho = 0.644 Χ^2^ = 0.095
*Questionário de Atividades de Autocuidado com o Diabetes* (QAD)^( 19 )^	- General food - Specific food - Physical activity - Blood glucose monitoring - Foot care - Medication use - Smoking 17 items	Likert	Version Brazilian of SDSCA	Cronbach’s alpha = 0.86. Correlation: r = 0.29 -1.00
Questionnaire on skills and self-care activities of patient with diabetic foot^( 20 )^	- Personal data and relatives - Self-care capacity - Foot self-care 20 items	Likert	Peruvian Spanish	- Missing from the article
The social-support scale for self-care in middle-aged patients with type 2 diabetes (S4-MAD)^( 21 )^	- Social support for nutrition activities - Physical activity - Blood glucose Self-monitoring - Foot care - Smoking 30 items	Alternative (yes/no)	Iran English	Cronbach’s alpha = 0.94 Correlation: Χ 2: 2.03 R = 0.87 (intraclass) KMO = 0.92
*Questionário de Atividades de Autocuidado com os Pés para Diabéticos* (QPED)^( 22 )^	- Health service - Social support - Foot self-care compliance 14 items	Alternatives	Portuguese	CVI = 0.86 Cronbach’s alpha =0.76
*Questionário do Comportamento Planeado na Diabetes – Cuidado Com os Pés* (QCP-CP)^( 23 )^	- Intentions (2) - Attitudes (5) - Subjective standards (3) - Behavioral control perceived (4) - Action planning (4) - Coping planning (4) 22 items	Scores	Spanish	Cronbach’s alpha = 0.675 CCI=0.675 KMO: 0.741
Diabetes foot self-care behavior scale (DFSBS)^( 24 )^	- Foot inspection - Washing - Drying - Finger inspection - Lotion application - Shoe use 7 items	Likert and alternatives	Chinese	Cronbach’s alpha = 0.73 Kappa: 0.87 Correlation: Rho =0.87 R= 0.45
Frankfurter Catalogueof Foot Self-Care – Prevention of the Diabetic Foot Syndrome (FCFSP)^( 25 )^	- Professional assistance in foot care - Self-control of the feet - Self-control of shoes and socks - 19 items	Likert	German	Cronbach’s alpha = 0.84
Foot Care ConfidenceS cale (FCCS)^( 26 )^ Foot Care ConfidenceS cale/Foot-Care Behavior (FCCS-FCB)^( 27 )^	- Self-efficacy (12) - Preventive self-care behaviors (9) - Risky self-care behaviors (8) 29 items	Likert and alternatives	English Mexican version of FCCS	CVI: 1 α = 0.92 KMO = 0.758
The diabetic foot self-care questionnaire of the University of Malaga, Spain (DFSQUMA)^( 28 )^ The diabetic foot self-care questionnaire of the University of Malaga, French (DFSQ-UMA Fr)^( 29 )^	- Foot care - Shoe care - Sock care 16 items	Likert	Spanish Version in French	CVI >0.75 Cronbach’s alpha = 0.89 Kappa = 0.84 Correlation: r = 0.34 KMO: 0.89 Cronbach’s alpha = 0.922 Kappa = 0.84 Correlation: r = 0.48 (items) r = 0.89 (intraclass) KMO = 0.89
Diabetes Foot Diseaseand Foot Care (DFDFC-Q)^( 30 )^	- Diabetes-related foot diseases - Relaxation - Foot self-care - Shoes - Foot care education - Professional foot care 12 items	Likert	English	Cronbach’s alpha =0.672
Modified Diabetic FootC are Knowledge (MDFCK)^( 31 )^	- DM management (5) - Prevention of foot injuries (2) - Foot condition (2) - Foot hygiene (3) - Appropriate footwear (2) - Toenail care (1) 15 items	Alternative: correct/wrong	Indonesia	Internal consistency Kr-20: 0.75
Modified Diabetic Foot Care Behaviours (MDFCB)^( 31 )^	- General DM management (4) - Foot condition (4) - Foot hygiene (4) - Shoes (11) - Foot moisturizer (2) - Nail care (5) - Prevention of foot injuries (1) - Treatment of foot injuries (3) 34 items	Score	Indonesia	Cronbach’s alpha = 0.81.

*CVI - Content Validity Index; r - Pearson’s r; rho - Spearman’s rho; χ^2^ - Chi-Square; KR – Kuder-Richardson; KMO - Kaiser-Meyer-Olkin; DM – Diabetes Mellitus.*

## DISCUSSION

The foot self-care of people with diabetes is a priority within the health scenario, considering the prevention of complications that can be avoided with this practice. The economic, physical and psychosocial consequences caused by this complication can be avoided by performing a good foot assessment, which can be mediated by validated instruments^( 32 )^.

However, this is not an easy task, mainly because it involves diabetes and its complexity, which requires the necessary attention to meet the multidimensionality of care^( 2 )^. It is noticeable that research in the literature seeks to meet this demand, due to the fact that we have found a considerable number of studies in the area, but which, if compared, would not be able to present in a single instrument with all the facets that involve care for children with DFU from prevention to treatment.

Synthesizing our evidence, we highlight that foot self-care should involve inspection, hygiene, washing and drying, lotion use, and sock and shoe care^( 33 )^. A review study showed results similar to ours, in which people with DFU knew to some degree about foot inspection care, foot hygiene, blood glucose control and foot protection. However, such knowledge was not properly applied in practice, revealing a common deficiency that is much discussed and revealed in the literature^( 34 )^.

Foot self-care measures seem simple on the one hand, but in practice they become complex. There are a series of instruments used to assess DFU placements. However, even in clinical practice, this becomes a challenge, pointed out by professionals themselves, as there are no instruments that are applicable to the general population and that respect the many aspects involved, such as sociodemographic, behavioral and beliefs issues^( 1 , 35 )^. When care, such as feet and finger inspection, cleaning, shoe and sock care, is not put into practice, due to lack of professional guidance or a person’s own choice, risk behaviors are established that make self-care difficult and favor DFU appearance.

Having a family support network and educational knowledge about self-management is essential to prevent DFU and improve the quality of life of people with this condition. Emotional support, which can be found in the family or in the professional, is presented as a pillar to strengthen self-care measures, and should also be concerned together with the person, with the correct management of diabetes, which is closely linked to this clinical condition^( 36 )^.

We also emphasize that DFU is a palpable problem in clinical reality and deserves to be better explored. In our findings, only one study was quasi-experimental, in which it was possible to carry out an intervention and assess knowledge and behavior regarding foot care before and after implementing the intervention. This leads us to reflect that more interventions and application of these instruments are needed in order to improve people’s knowledge about foot self-care^( 34 )^.

In addition, the construction of instruments for clinical practice must be built assertively, respecting the methodological aspects recognized in the literature. In the present review, few instruments were clearly presented: some omitted important information from statistical tests and others were not presented in studies that prove their practical use. It is important to include comparative studies, as reinforced by the recent systematic review^( 37 )^, in which the combined foot self-care scores resulted in 62.84%. This value was higher than in studies that compared two groups of people with DM1 and DM2 compared to groups that had DM2, corroborating the importance of not only building instruments, but systematizing their application in practice. The literature addresses that topics related to foot self-care revolve around self-care knowledge, the high costs that can trigger with DFU, in addition to some barriers and resistance to performing this self-care.

Nail care, prevention and treatment of foot injuries are also strengths that must be recognized and analyzed as well presented in our review. As in our study, the instruments address dimensions related to foot self-care, but bring some items related to DM management, which are important in the context of foot care, given that it is essential to bring completeness to this care. In line with this knowledge, it is scientifically proven that the process of health education in various self-management interventions has a positive impact on the behavior and self-efficacy of people with diabetes in terms of performing foot self-care^( 38 )^. Furthermore, regular inspection and examination of at-risk feet can prevent secondary injuries and complications, making it an essential part of diabetes management^( 39 )^.

According to the International Working Group on the Diabetic Foot (IWGDF), there are five key elements to prevent DFU, namely: (1) identification of the at-risk foot; (2) regular inspection and examination of the at-risk foot; (3) education of patient, family and healthcare providers; (4) routine wearing of appropriate footwear; and (5) treatment of pre-ulcerative signs. This care allows an early identification of the alterations present, promoting a timely treatment and avoiding further complications^( 40 )^.

Health education has several advantages and does not overlap with other clinical activity, strengthening compliance and encouraging self-care. Moreover, it must consider each person’s conditions, respecting their individuality and reality^( 39 )^. It is advisable to assess whether this person, family member or caregiver has understood the messages and is motivated to act and comply with the guidelines, to ensure sufficient self-care skills. A properly trained team of health professionals should address the five key elements to preventing DFU^( 41 )^.

The importance of a health team is highlighted, especially in primary care, which has within its guidelines the role of developing prevention and health promotion actions for people with DM^( 33 )^. However, there is an overload on the essential functions of this service, which can compromise some care, such as supervision and self-care assessment. It is also noteworthy that nursing is the professional category that has a large number of actions aimed at preventing this complication and that it is presented as a reference in DFU prevention and care^( 33 )^.

### Study limitations

As limitations of this study, we highlighted that some instruments were not presented, and it was not possible to identify the original version of the same, referring to a gap in the publication of these studies. Due to the methodology adopted, deeper concepts about foot self-care could not be better explored, given the objective followed.

### Contributions to nursing, health, or public policies

The main contribution of this study is to provide professionals with knowledge of instruments for assessing the foot self-care of people with diabetes available in the literature so that they can apply them in their clinical practice, according to each one’s needs and reality.

We encourage new studies in the area so that self-care measures with DFU become an increasingly constant and applicable reality among health practices.

## FINAL CONSIDERATIONS

We identified 16 foot self-care assessment instruments, 11 of which are specific to foot care, one of which was built and validated in Brazil. Self-care, due to its complexity, also presented itself in a different way, linked to social support, knowledge, modified behaviors, compliance and frequency of care delivery. In general, the instruments considered foot inspection, hygiene, hydration, sock and shoe use, in addition to shoe inspection care, essential self-care measures.

Considering the findings, it is possible to verify a considerable number of studies that report the need for foot self-care, although still incipient in clinical practice. It is also suggested the cross-cultural adaptation and use of these instruments in practice and research, in order to obtain useful data that facilitate decision-making and that are really disseminated by all health professionals.
