# ERRATUM

**DOI:** 10.1590/0034-7167.20237604e03

**Published:** 2023-11-10

In the article “Diabetic foot ulcer self-care assessment: a scoping review”, with DOI number: https://doi.org/10.1590/0034-7167-2022-0555, published in Revista Brasileira de Enfermagem, 2023;76(3): e20220555, in the title:

Where it read:

**Diabetic foot ulcer self-care assessment: a scoping review**

*Avaliação do autocuidado da úlcera do pé diabético: revisão de escopo Evaluación del autocuidado de la úlcera del pie diabético: revisión del alcance*

It reads:

**Instruments for assessing foot self-care of people with diabetes: a scoping review**

*Instrumentos de avaliação do autocuidado com os pés de pessoas com diabetes: revisão de escopo*

*Instrumentos para evaluar el autocuidado con los pies de personas con diabetes: revisión del alcance*

